# Langerhans cells in hypospadias: an analysis of Langerin (CD207) and HLA-DR on epidermal sheets and full thickness skin sections

**DOI:** 10.1186/s12894-019-0551-8

**Published:** 2019-11-12

**Authors:** Bernhard Haid, Daniela Reider, Felix Nägele, Anne-Françoise Spinoit, Elisabeth Pechriggl, Nikolaus Romani, Helga Fritsch, Josef Oswald

**Affiliations:** 1 Department of Pediatric Urology, Hospital of the Sisters of Charity, Ordensklinikum Linz, Seilerstätte 4, 4020 Linz, Austria; 2 Department of Urology, Ludwig Maximilians University, Marchioninistraße 15, 81367 Munich, Germany; 3 Department for Dermatology and Venereology, Medical University Innsbruck, Anichstraße 35, 6020 Innsbruck, Austria; 4 Section for clinical and functional Anatomy, Medical University Innsbruck, Müllerstraße 59, 6020 Innsbruck, Austria; 5 Department of Urology, University Clinic Gent, Corneel Heymanslaan 10, 9000 Gent, Belgium; 6 Department of Plastic, Reconstructive and Aesthetic Surgery, Medical University Innsbruck, Innerkoflerstraße 1, Innsbruck, Austria

**Keywords:** Langerhans cells, Hypospadias, Foreskin, HPV, HIV

## Abstract

**Background:**

Hypospadias are among the most common genital malformations. Langerhans Cells (LCs) play a pivotal role in HIV and HPV infection. The migration of LC precursors to skin coincides with the embryonic period of hypospadias development and genetic alterations leading to the formation of hypospadias impact the development of ectodermally derived tissues. We hypothesized that this might be associated with a difference in frequency or morphology of epidermal and dermal LCs in hypospadias patients.

**Methods:**

A total of 43 patients from two centers were prospectively included into this study after parental consent and ethics approval. Epidermal and dermal sheets were prepared from skin samples of 26 patients with hypospadias, 13 patients without penile malformations and 4 patients with penile malformations other than hypospadias. Immunofluorescence staining of sheets was performed with anti-HLA-DR-FITC and anti-CD207/Langerin-A594 antibodies. Skin sections from 11 patients without penile malformation and 11 patients with hypospadias were stained for Langerin. Frequencies as well as morphology and distribution of epidermal and dermal LCs on sheets and sections were microscopically evaluated. Cell counts were compared by unpaired t-tests.

**Results:**

There was no difference in frequency of epidermal LCs, Neither on sheets (873 ± 61 vs. 940 ± 84LCs/mm^2^, *p* = 0.522) nor on sections (32 ± 3 vs. 30 ± 2LCs/mm^2^, *p* = 0.697). Likewise, the frequency of dermal LCs (5,9 ± 0,9 vs. 7.5 ± 1.3LCs/mm^2^, *p* = 0.329) was comparable between patients with hypospadias and without penile malformation. No differences became apparent in subgroup analyses, comparing distal to proximal hypospadias (*p* = 0.949), younger and older boys (*p* = 0.818) or considering topical dihydrotestosterone treatment prior to surgery (*p* = 0.08). The morphology of the LCs was not different comparing hypospadias patients with boys without penile malformations.

**Conclusions:**

LCs are present in similar frequencies and with a comparable morphology and distribution in patients with hypospadias as compared to children without penile malformations. This suggests that patients with hypospadias are not different from patients with normal penile development considering this particular compartment of their skin immunity.

## Background

Hypospadias affects 1 in 125–300 male births, representing the second most common genital malformation after undescended testes [1]. The malformation of the penis present in hypospadias, however, does not only involve the urethral opening being situated abnormally at the ventral side of the penis. All tissues including skin, notably on the ventral aspect of the penis, seem underdeveloped and altered in appearance. The events related to the formation of hypospadias happen in very early phases of the genital tubercle outgrow starting in gestational week 8; differentiation of the urethral epithelium is determined by mesenchymal signaling [2]. Thereby, not only the organogenesis of the urethra is defined but also prepuce morphogenesis and surface epithelia of ectodermal origin are influenced [3, 4].

Except concerning hormonal receptors, the repercussions of these early fetal events on other penile tissues have not yet been adequately investigated [3, 5].

Langerhans cells (LCs), the dendritic cells of epithelia, are dispersed in the epidermis as a dense network of immunologic gatekeepers, internalizing possible pathogenic antigens and presenting them to T-cells in draining lymph nodes, thereby initiating and regulating the specific immune response [6]. They are critically involved in human immunodeficiency virus (HIV) and human papillomavirus (HPV) infection [7, 8]. The typical hallmark of human LCs, as opposed to other dendritic cells, is the expression of Langerin (CD207), a C-type lectin serving as pathogen receptor and comprising the archetypical Birbeck granules [9]. LCs develop and seed the epithelial tissues already during embryogenesis, notably in the very same period when later hypospadias are determined [10]. Differences in function of these specialized immune cells could implicate altered susceptibility to HIV or HPV.

Despite these important implications in the skin immune system, the presence or morphology of Langerhans Cells have never been investigated in patients with hypospadias up to now as to our knowledge.

We hypothesized that due to developmental differences in epithelial embryogenesis, the frequency or morphology and consequently also the function of epidermal and dermal LCs in patients with hypospadias might be altered in comparison to patients without a penile malformation.

## Methods

The study protocol has been approved by the ethics committee of the Hospital of the Sisters of Charity (EK22/14). After informed consent of all parents a total of 43 patients (Linz/Ghent 32/11) were prospectively included.

**Fig. 1:**
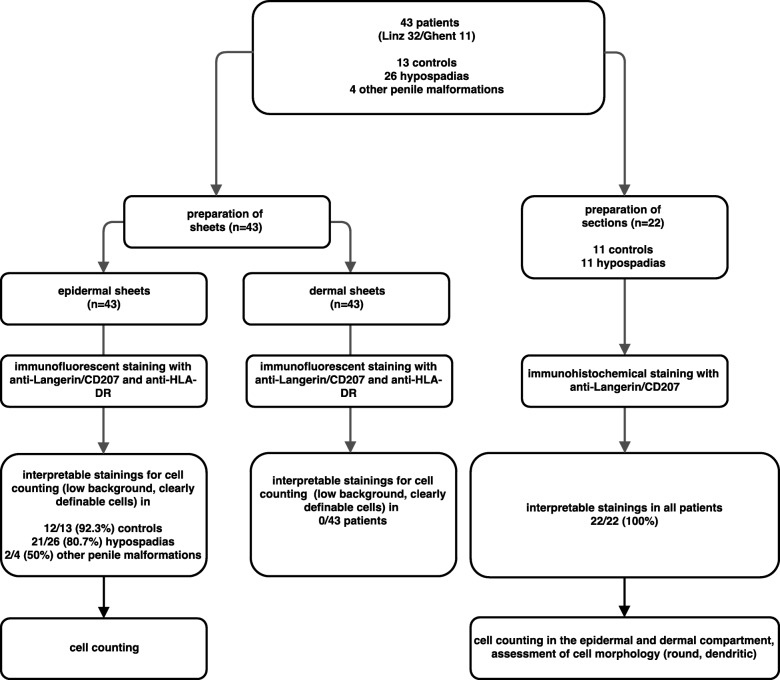
Flowchart displaying the allocation of patients to the two methods and the respective staining protocols

During surgery, small pieces (7-10 mm^2^) of whole preputial skin from lateral parts of the prepuce were obtained. Any sign of potential inflammation, for instance balanoposthitis or a clinical suspicion of Lichen Sclerosus as well as a history of balanoposthitis resulted in non-inclusion of the patient. In course of analyzing the samples, all section stainings were checked for histological signs of inflammation (dermal leucocyte infiltration) to subsequently exclude them in order to preclude a potential interference with our results. The preparation steps for the two different methods applied are detailed below. The allocation of specimens to the different methods is displayed in Fig. 1.

Patients’ characteristics are presented in Table 1.

**Table 1:** Patients characteristics

	all patients (*n* = 43)	Linz (*n* = 32)	Gent (*n* = 11)	p
age at surgery mean/median [months]	49 / 15	36 / 15	26 / 14	0.4^a^
no penile malformation (%)	13 (30.2%)	12 (37.5%)	1 (9.1%)	
hypospadias	26 (60.4%)	19 (59.4%)	7 (63.6%)	
distal hypospadias (% of all hypospadias)	16 (61.5%)	11 (57.9%)	5 (71.4%)	
proximal hypospadias (% of all hypospadias)	10 (38.5%)	8 (42.1)	2 (28.6%)	
thereof with preoperative androgen treatment (local DHT^b^ for 6 weeks until 4 weeks before surgery)	7 (70%)	7 (87.5%)	0	
other penile malformations	4 (9.3%)	1 (3.1%)	3 (27.2%)	
buried penis	2 (50%)	0	2 (66.6%)	
epispadias	2 (50%)	1 (100%)	1 (33.3%)	

^a^students t-test ^b^ Dihydrotestosterone, applied twice daily

In patients with proximal hypospadias and a glans diameter of less than 14 mm, topical dihydrotestosterone 2.5% gel (Andractim®) was applied over a period of 6 weeks until 4 weeks prior to surgery twice daily.

### Sheets

**Fig. 2:**
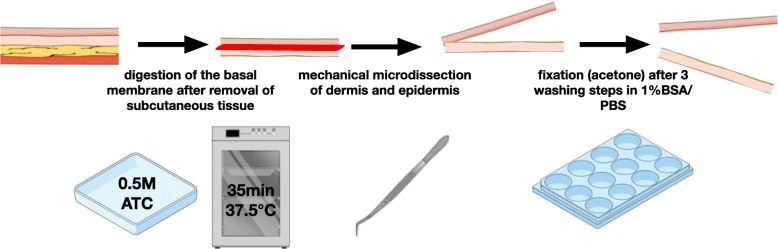
Schematic display of epidermal / dermal sheet preparation technique from human full skin. ATC, ammoniumthiocyanate

Immediately after harvesting, skin was freed from sub-epithelial fatty tissue and conserved in a sterile PBS/1%BSA (phosphate buffered saline / 1% bovine serum albumine) solution. At the latest 2 h thereafter, the skin was floated – dermal side down – on 0.5 M ammoniumthiocyanate solution for 35 min at 37 °C. The epidermis was then peeled off the dermis using microsurgical instruments. After fixation (synthesis grade acetone, 20 min at room temperature) and three washing steps using PBS/1%BSA, the dermal and epidermal sheets were cut into small pieces (4-5 mm^2^), frozen in a drop of PBS/1%BSA and stored at − 20 °C [11]. A detailed protocol of sheet preparation is provided in Fig. 2.

After thawing, epidermal as well as dermal sheets were incubated with an unconjugated primary antibody (anti-human CD207/Langerin; clone MB22-9F5, mouse IgG1, Miltenyi Biotec, Bergisch Gladbach, Germany) for 1 h at 37 °C, followed by three 10-min washing steps. Subsequently the sheets were incubated with a fluorochrome-coupled secondary antibody (Alexa-Fluor 594-conjugated, isotype-specific goat anti-mouse IgG1, Invitrogen/Thermo-Fisher) for 1 h at 37 °C, again followed by three 10-min washing steps. The staining sequence was extended by incubating the sheets with an excess concentration of mouse IgG (100 μg/ml)) to quench residual free binding sites of the preceding anti-mouse Ig antibodies. Finally, sheets were incubated with anti-human HLA-DR (clone L243, mouse IgG2a, BioLegend) followed by Alexa-Fluor 488-conjugated, isotype-specific goat anti-mouse IgG2a, Invitrogen/Thermo-Fisher) for 1 hour each at 37 °C. For nuclear staining, dermal sheets were incubated in DAPI dilactate (Invitrogen® / Molecular Probes). For each antibody, an isotype control staining was performed to exclude unspecific staining results. Immunolabeled specimens were mounted in Vectashield mounting medium (Vector Laboratories, Burlingame, CA) and analyzed on a conventional fluorescence microscope (Olympus BX60).

Only when an unequivocal epidermal keratinocyte structure (“cobblestone pattern”) could be seen in transmission light, stainings were considered valid. Due to the thickness of the dermal part of the sheets, reproducible cell counting in the dermis was not possible using sheets and conventional fluorescence microscopy.

On every epidermal sheet, HLA-DR+ and langerin+ cells cells were counted in 12 high power fields (40× objective lens magnification). Density of cells/mm^2^ was calculated by means of a calibrated micrometer slide.

### Sections

Immediately after harvesting, full skin of 22 patients (Fig. 1) was fixed in 4% formaldehyde (2–4 h) and then immersed in paraffin blocks for conservation and transport. 5 μm whole skin sections were prepared on a rotation microtome and mounted on SuperFrost® Plus slides. The sections were stained using anti-human CD207/Langerin (clone 929F3.01 undiluted hybridoma cell supernatant, rat IgG2a, gift of Dr. S. Saeland and Dendritics, Lyon, France) followed by immunohistochemical labeling (Ventana universal secondary antibody, biotinylated IgG anti-goat/mouse/rat; Ventana ultra view DAB-MAP detection kit) using a standardized, automatized staining technique (DAP-MAP IHC discovery research standard protocol, Ventana, Roche®).

LCs were counted in 4 epidermal and 4 dermal fields that were placed randomly measuring 100x100μm/section and patient. Counting and measuring was performed using the Image J Software (National Institute of Health, Bethesda, ML, USA).

LC morphology was classified into round and dendritic shaped and documented alongside cell counting, specifying the relative proportions of round and dendritic shaped LCs.

### Statistics

Cell counts for epidermal and dermal LCs on sheets as well as on sections were compared by unpaired t-tests. The cut-off for the alpha error was defined as < 0.05, for multiple testing a Bonferroni correction was applied. Data analysis and figure design was performed using Prism 6.0 (Graphpad Software, Inc., San Diego, CA, USA).

## Results

### Sheets

Interpretable staining without signs of inflammation (dermal leucocyte infiltration) could be achieved in samples from 12 patients (92.3% of all included patients) without penile malformations, 14 (87.5%) with distal hypospadias, 7 (70%) with proximal hypospadias, one with buried penis (50%) and one with epispadias (50%) translating into a total of 81.4% of all epidermal sheets prepared being amenable for analysis. HLA-DR/CD207 double positive cells were considered LCs.

**Fig. 3:**
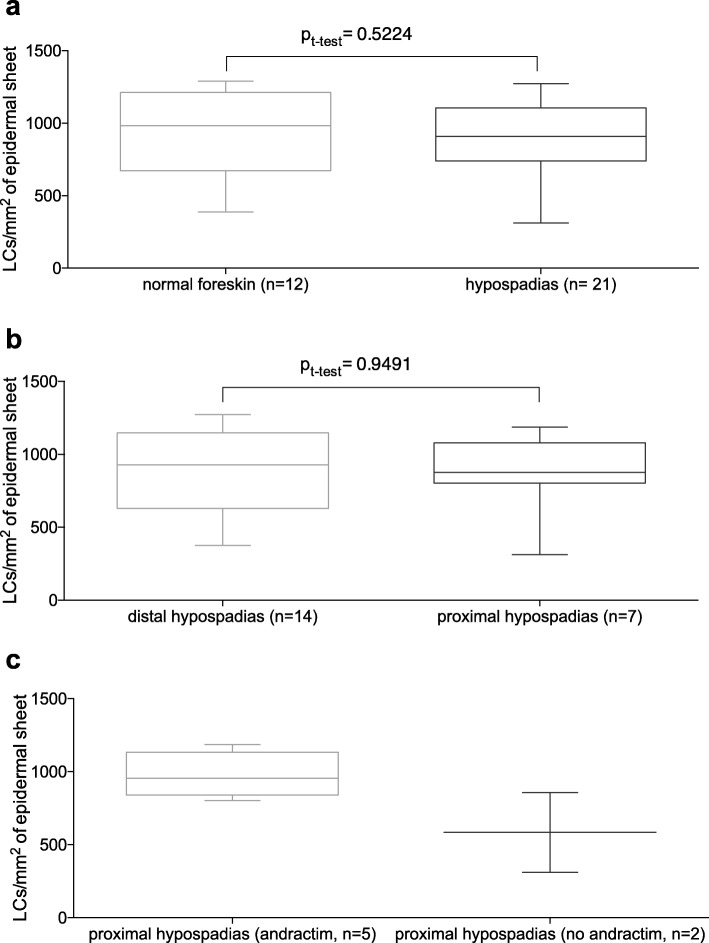
Box-plots of mean Langerhans cell (LC) counts per mm^2^ on immunofluorescence stained epidermal sheets comparing **a** patients without penile malformations to all included patients with hypospadias, **b** patients with distal hypospadias to patients with proximal hypospadias, and **c** patients with proximal hypospadias who did not receive topical dihydrotestosterone (DHT, andractim) before surgery to those who did receive 6 weeks of DHT until 4 weeks prior to surgery. Error bars represent min-max

**Fig. 4:**
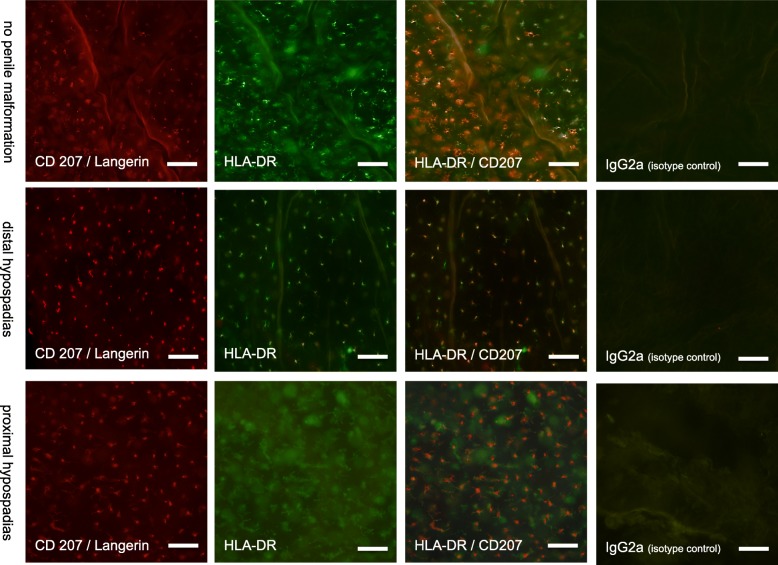
Immunofluorescence stainings with anti-HLA DR and anti-CD207 / Langerin as well as IgG2 isotype controls of epidermal sheets. Scale bar represents 60 μm

The mean frequency of epidermal HLA-DR/CD207 double positive cells per mm^2^ accounted to 873.4 ± 61.6 in patients with hypospadias (*n* = 21) as compared to 940.2 ± 84.2 in patients without penile malformation (*n* = 12, *p* = 0.522, t-test). There was no significant difference in LC frequency between distal and proximal hypospadias (876.2 ± 78.7, *n* = 14, distal vs. 867.6 ± 105.5, *n* = 7 proximal, *p* = 0.9491). Comparing patients with proximal hypospadias who had received topical dihydrotestosterone prior to surgery to those who had not, we found no significant difference in epidermal LC density (585.5 ± 273.1, *n* = 2 vs. 980.4 ± 68.9, *n* = 5, *p* = 0.0857). The morphology of the epidermal LCs was the same in all samples Figs. 3 and 4.

**Fig. 5:**
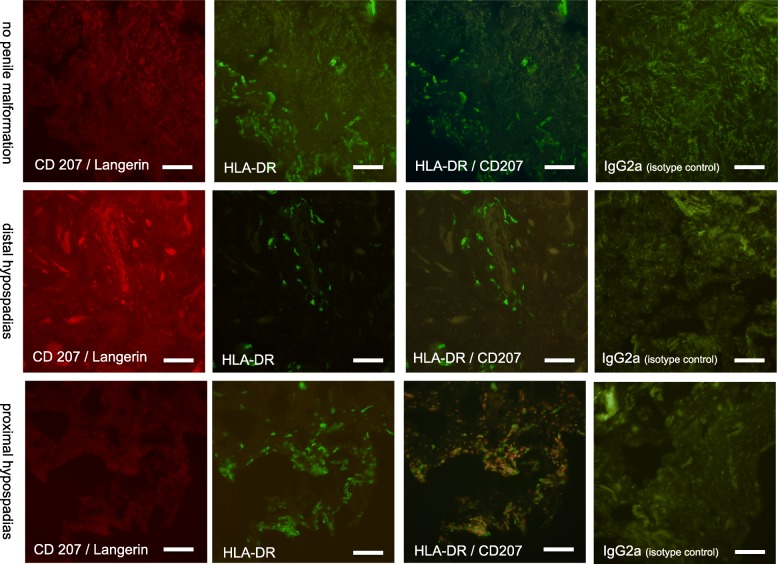
Immunofluorescence stainings with anti-HLA DR and anti-CD207 / Langerin as well as IgG2 isotype controls of dermal sheets. Note that none of the HLA-DR+ cells depicted co-expresses Langerin. Scale bar represents 60 μm

Stainings of the dermal sheets revealed single Langerin-positive cells, however, due to background staining reliable and reproducible cell counting on these sheets was not possible with the available microscopic facilities. Figure 5 provides immunofluorescent images showing the dermal sheet staining.

**Fig. 6:**
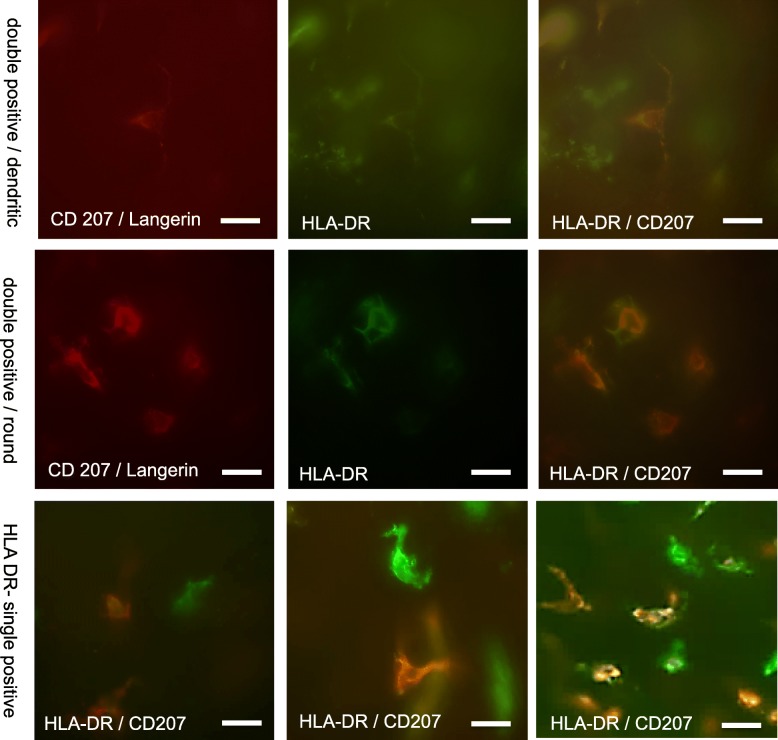
Morphology of epidermal LCs (dendritic, round). Note rare examples of HLA-DR single positive epidermal cells. Scale bar represents 10 μm

The morphology of the epidermal LCs was comparable in all samples**.** Most epidermal LCs were dendritically shaped, however, some (10–15%) were rounded. No conspicuous and consistent differences could be detected in comparison with LCs from healthy foreskin. While most epidermal HLA-DR-positive cells were also Langerin positive, on some sheets we found areas with intermingled HLA-DR single positive cells. Sample images of single Langerhans cells and their morphology, as defined for this project, are shown in Fig. 6.

**Fig. 7:**
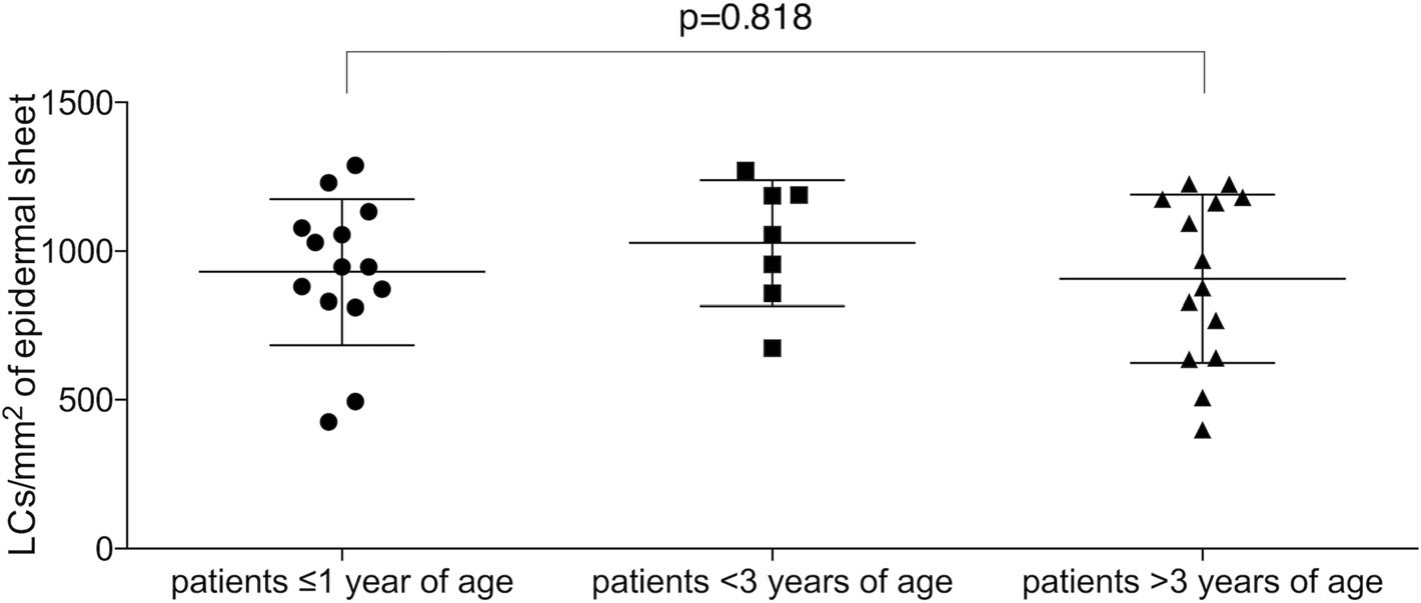
Dot-plots of LC frequency of patients aged ≤1 year, between 1 and 3 years and < 3 years. Lines represent mean with standard deviation

LC frequency accounted to 930.3 ± 65.61 (*n* = 14) in patients < 1 year of age, 1028 ± 80.26 (*n* = 7) in patients between 1 and 3 and 907.0 ± 75.65, (n = 14) in patients over 3 years of age (*p* = 0.818 < 1 vs. > 3 years of age) Fig. 7.

The patient with buried penis had a mean LC count of 880.3/mm^2^. In the single interpretable staining of a patient with epispadias we found 1055.79 LCs/mm^2^.

### Sections

On all stained sections (*n* = 22, 11 hypospadias, 11 patients without penile malformation), clearly evaluable staining results were achieved. Dermal as well as epidermal LC density was assessed. CD207-positive cells were considered LCs.

In the epidermis, we found a mean of 31.8 ± 2.7 LCs per mm^2^ (*n* = 11) in samples from patients without penile malformation as compared to 30.5 ± 1.9 (*n* = 11) in samples from patients with hypospadias (*p* = 0.697). In patients with distal hypospadias the LC frequency was similar as compared to those with proximal hypospadias (28.2 ± 2.1, *n* = 7 vs. 34.4 ± 3.1, *n* = 4, *p* = 0.13).

**Fig. 8:**
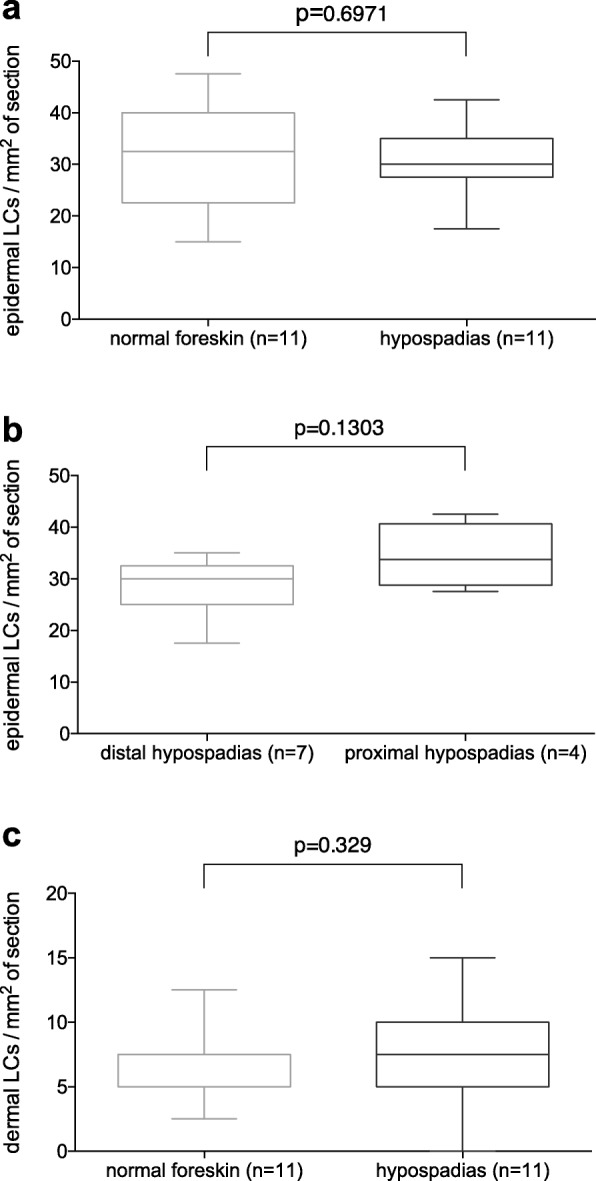
Box-plots of mean Langerhans cell (LC) counts per mm^2^ on immunohistochemically stained full skin sections comparing **a** patients without penile malformations to all included patients with hypospadias, **b** patients with distal hypospadias to patients with proximal hypospadias, and **c** dermal LC counts per mm^2^ in patients without penile malformations to all included patients with hypospadias. Error bars represent min-max

**Fig. 9:**
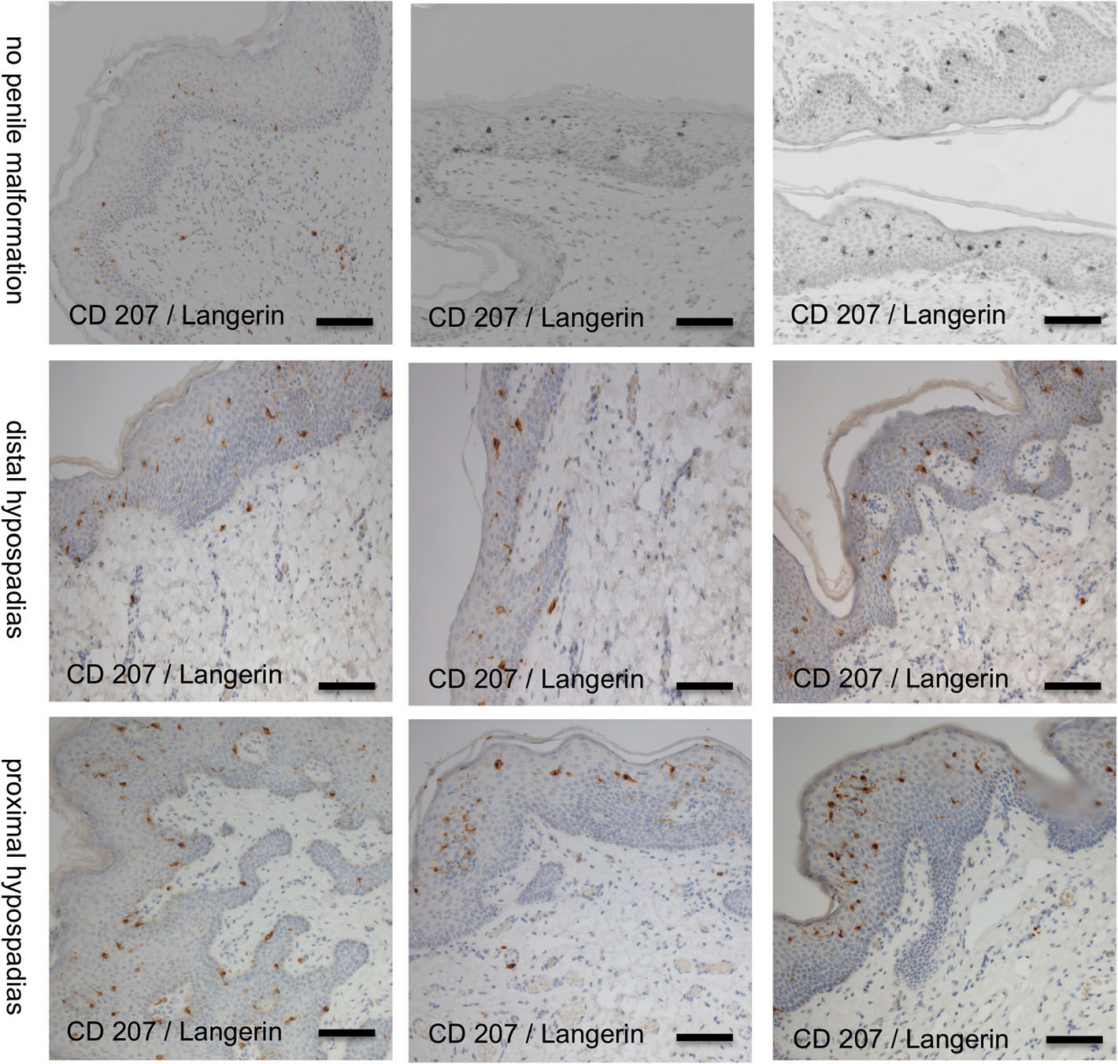
Immunohistochemically stained full skin sections with anti-Langerin (929F3.01). Scale bar represents 100 μm

In the dermis a mean LC frequency per mm^2^ of 5.9 ± 0.9, n = 11 was assessed in patients without penile malformations whereas the LC frequency in hypospadias patients accounted to 7.5 ± 1.3, n = 11 (*p* = 0.329) Figs. 8 and 9.

Morphology was comparatively analyzed on samples of about 100 LCs each in epidermis and dermis. 8.4% of all epidermal LCs were round as compared to 91.6% with a dendritic appearance. In contrast, virtually all (99.1%) dermal CD207/Langerin+ cells had a round shape. There was no difference in the proportion of shapes of LCs comparing patients with and without penile malformations.

## Discussion

By thorough evaluation using two different methods our data demonstrate that LCs are present in patients with hypospadias in the same frequency and morphology as in foreskin from children without penile malformation. Consequently, we assume that their ontogeny is not disturbed and we hypothesize their immunological function is preserved in hypospadias patients.

The relevance of this finding – negative data – becomes apparent in view of the altered (fore) skin embryology and the changes to skin function in hypospadias patients as well as when considering the role of LCs in the foreskin in sexually transmitted diseases, notably HIV and HPV. Both are not only very common but also associated with a massive burden of morbidity.

Hypospadias are linked to early embryological changes at time of outgrow of the genital tubercle starting from gestational week 8, when mesenchymal signaling, notably involving FGF, which is required for the outgrow of the genital tubercle, determines epithelial differentiation. Mesenchymal FGF signaling has been shown to affect ectodermal as well as endodermal epithelia [4]. In a murine model, ectodermal deletion of FGFR2 resulted in the most severe hypospadias with major alterations to the foreskin, highlighting a major role of FGFR2 in the developing genital surface epithelia [3]. Various changes in skin development and marker allocation have been shown to occur as a consequence [2, 12]. Foreskin derived fibroblasts from children with hypospadias react differently to endocrine stimuli, possibly based on genetic variation in these subjects [13]. Furthermore, a subcutaneous muscle layer, termed the “dartos fascia” has been shown to be disorganized in patients with hypospadias [14]. This evidence highlights the impact of early fetal events during the outgrow of the genital tubercle on the development of penile skin in boys with hypospadias.

Androgen and estrogen receptors as well as molecules responsible for their regulation, notably ZEB1, are expressed differently in skin of hypospadias patients [5, 15]. Interestingly, ZEB1 has also been implicated in the regulation of the fate of CD8^+^ T-cells [16] and has been shown to be relevant in LC maturation [17]. This points at a possible relationship between androgen receptor regulation being involved in the embryology of hypospadias and the functioning of the specific immune system – including LCs.

LCs acquire their typical immunophenotye gradually during fetal life and after birth. It has been shown in a mouse model, that Langerin is expressed only after birth [18]. Another prototypical marker of LCs, the NF**-κ**B activator RANK, is already expressed by the end of the second trimester in adult like levels [19]. LC precursor cells, however, are present in the dermis as early as gestational week 9–14 [20]. This matches the timeframe, when – at the level of the genital tubercle – changes become effective that ultimately lead to the formation of hypospadias [1]. Consequently, finding a frequency of LCs unchanged compared to patients without penile malformation, might be interpreted as evidence for an unhindered colonization of the penile skin with immune precursor cells in early fetal life. Our findings would suggest that the penile skin epithelium might be less affected by the underlying genetic changes, at least with regard to the skin-resident Langerhans cells.

LC frequency in human foreskin as well as in the glandular epithelia has been assessed multiply, notably in connection with HIV research. The LC frequency in vaginal and foreskin epithelium was found to be high, comparable to our results. Also, an age related difference in LC frequency in foreskin epidermis has been described [21]. In our study, we could not observe different LC frequency dependent on patients’ age.

LCs are critically involved in HIV infection, with the removal of the foreskin leading to a reduced probability of female to male transmission from 2.49 to 1.18% [7, 22]. Independent from LC frequency also the polarity of exposition was found to modify the probability of infection with the inner foreskin being more susceptible to HIV infection [23]. Our findings might be interpreted as indirect evidence that males affected by hypospadias carry the same risk of HIV infection compared to those who have no genital malformation. However, lacking a direct proof this remains hypothetical but fits the lack of reports about excess HIV infection rates in hypospadias patients. Removal of foreskin during hypospadias surgery implies the same relative protection in hypospadias patients as compared to others.

HPV infects basal epithelial cells [24]. The presence of foreskin is related to the likelihood of transmission from men to women as well as the likelihood of persistence of infection [25]. Unlike in HIV infection, LCs play a critical role in modifying the response of the immune system to the HPV infection. The presence of T regulatory cells – presumably a result of lacking co-stimulation by LCs - in HPV associated cervical cancer lesions has been shown to probably be associated to worse outcomes [26]. Furthermore LCs are critically involved in rendering T-cells unresponsive and thereby allowing for HPV progression [8]. Penile cancer is relatively rare and often associated with HPV. Additionally, a role in the inhibition of cutaneous carcinogenesis has been attributed to LCs in a murine model for squamous cell cancer [27].

Like for HIV, our finding of an unchanged frequency of LCs in hypospadias patients points at a risk of HPV infection similar to that of a non-hypospadias population, however, without direct proof.

Preoperative hormonal treatment is commonly used, mainly for proximal hypospadias to increase glandular diameter and penile girth in order to prevent postoperative complications. It has been shown in an animal model, that androgen stimulation induces topical inflammation, what could be relevant to LC activation and migration [28]. The lag time between androgen application and surgery in our patients was at least 4 weeks whereas there was no “testosterone holiday” in the above-mentioned animal model. Furthermore, we used topical dihydrotestosterone whereas in the cited study, intramuscular testosterone was applied. In our patient cohort, 5/7 proximal hypospadias received a preoperative topical treatment with dihydrotestosterone ointment. As there was no difference in LC frequency, we conclude that preoperative androgen application – with a “testosterone holiday” of 4 weeks and topical administration – does not prompt LC migration by a topical inflammatory response.

Studying potential repercussions of embryological events in human tissues implicates a number of limitations. Prenatal sampling of skin is unprecedented in humans; such research would require an animal model, as to our ends also for genital malformations. We are limited to describing the state of LCs after birth, however, we cannot draw any conclusions on whether the ontogeny and the colonization of skin by these cells during embryological developments is as normal as their appearance in our samples. Moreover and most importantly, the lack of a functional evaluation of the LCs, e.g. by induction of inflammation prior to tissue sampling, that is not possible due to ethical concerns, limits the scope of our study. We therefore rely on the presence of physiological numbers of LCs as an indicator of their normal functioning. The dermal compartment, that is critical for LC traffic and the functioning of the immune system, could only be assessed on sections. With regard to the data on LC ontogeny and as elaborated above, however, the choice to investigate the frequency and anatomy of LCs after birth by sheet and section analysis seemed an important and logical step. In case of a disrupted embryological colonization, changes were to be expected [18].

In view of these limitations, the conclusions made herein are limited to a snap-reading of physiological LC frequency. With these findings relying on a meticulous sampling excluding any bias and two different methods for analysis (sheets and sections), however, the data itself seem undisputable. Furthermore this is the first report in literature to assess LCs in children with penile malformations.

For future research, refined techniques such as multicolour-flow-cytometry of skin cell suspensions or single-cell-transcriptome-analyses may reveal peculiarities of the immune system in the skin of hypospadias patients [29, 30]. Furthermore, animal models with inducted penile malformations, as for example FGF10 of FGFR2 knockout mice could help shedding more light onto LC ontogeny, postnatal animal models could address the question of LC functionality [31, 32].

## Conclusions

Langerhans cells are present in the foreskin of hypospadias patients in a normal frequency and morphology compared to patients without penile malformation. This might suggest that patients with hypospadias are not different from patients with normal penile development considering their specific skin immunity, at least considering the epidermal compartment. Functional aspects as well as the dendritic cell subset composition of the dermis remain to be studied.

## Data Availability

The datasets generated and/or analysed during the current study are available over Zenodo, https://zenodo.org/record/1323160#.XL4Vey9XbUI

## Abbreviations

A488: Alexa Fluorochrome 488 (green)
A594: Alexa Fluorochrome 594 (red)
BSA: bovine serum albumine
CD: cluster of differentiation
DAPI: 4′,6-Diamidin-2-phenylindol (blue)
HIV: Human Immunodeficiency Virus
HLA: Human Leucocyte Antigen
HPV: Human Papilloma Virus
ICH: Immunohistochemistry
LC: Langerhans Cell
PBS: phosphate buffered saline
